# Odor Hedonic Profile (OHP): a self-rating tool of everyday odors

**DOI:** 10.3389/fnins.2023.1181674

**Published:** 2023-08-04

**Authors:** Bontempi Charlotte, Jacquot Laurence, Brand Gérard

**Affiliations:** ^1^Université de Franche-Comté, Besançon, France; ^2^Centre des Sciences du Goût et de l'Alimentation, CNRS, Inrae, AgroSup Dijon, Université Bourgogne Franche-Comté, Dijon, France

**Keywords:** olfaction, hedonic valence, pleasantness, everyday odors, self-rating

## Abstract

Odor hedonic estimation (pleasant/unpleasant) is considered the first and one of the most important dimensions in odor perception. Although there are several published scales that rate odor hedonicity, most of them use odorants that induce biases related to stimulus properties or test conditions and make difficult clinical or industrial applications. Thus, this study aimed to propose a model of odor hedonic profile (OHP) based on 14 items related to everyday odors without stimulus. The OHP is a self-rating tool based on the hedonic estimate representation and allows the determination of specific profiles, i.e., “conservative,” “neutral,” “liberal,” “negative olfactory alliesthesia,” and “positive olfactory alliesthesia.” It can be useful in different contexts (e.g., food studies) and general pathologies (e.g., eating disorders) or pathologies with mood/emotional disturbances (e.g., depression).

## 1. Introduction

The internal representation of hedonic experiences cannot be directly measured and must therefore be inferred from subjects' responses, which are reported as descriptive or numerical data. In addition, these responses can sometimes be linked to other data such as behavioral, psychophysiological, neurophysiological, or functional magnetic resonance imagery (fMRI) measures. Theoretical and practical differences among hedonic scaling methods and potential advantages and disadvantages have been described by Lim (2011). In olfaction, hedonic estimation has long been considered an important dimension of odor perception (e.g., Moncrieff, 1966; Schiffman, 1974; Land, 1979). Thus, odor hedonic estimations are common tasks in both scientific (including clinical) and industrial contexts. However, tests and measurement conditions across studies appear to be weakly standardized in olfaction due to multiple specificities. Most of the tasks use (1) odorants that widely differ in quality such as the stimulus properties, i.e., natural or synthetic, concentration, trigeminal component, etc. (Brand, 2006; Vodicka et al., 2010; Auffarth, 2013); (2) different procedures of stimulus presentation (into a test tube or with an olfactometer, in a single sniff or *via* free breathing, etc.); and (3) different types of scales (Clepce et al., 2014). Moreover, it is well known that olfactory perception shows a strong intra-individual variability related to many factors such as age, physiological state (hunger level, medication, etc.), emotional state (stress, mood, etc.), or the general context of perception including the sequential presentation of odorants (Nakano and Ayabe-Kanamura, 2017) and cognitive modulation (De Araujo et al., 2005), leading to significant variability in hedonic estimates. In the same way, interindividual variability related to sex (Brand and Millot, 2001; Sorokowski et al., 2019; Bontempi et al., 2021), sensitivity (Bontempi et al., 2022a), or experience toward odorants also results in wide hedonic flexibility across people and populations (e.g., Bontempi et al., 2022b).

From a methodological point of view, several tools are usually used to assess odor hedonic perception. Among them, Likert scales are the most frequently reported in the literature and graduated from extremely unpleasant to extremely pleasant with values from −5 to +5 (e.g., Distel et al., 1999), from +1 to +10 (e.g., Coppin et al., 2010), from −2 to +2 ( e.g., Doty et al., 1984; Masago et al., 2001; Cumming et al., 2011), and even more. Visual analog scales are also frequently used to assess odor hedonic estimation in different contexts such as in the elderly population (e.g., Markovic et al., 2007), in pain tolerance (e.g., Prescott and Wilkie, 2007), in olfactory lateralization (e.g., Thuerauf et al., 2008), in depression (e.g., Clepce et al., 2010), or in cancer chemotherapy (e.g., Ishinaga et al., 2018). Moreover, there are several questionnaires to assess general hedonicity, such as the Temporal Experience Pleasure Scale (Gard et al., 2006) or the Self-Assessment Anhedonia Scale (Olivares et al., 2005), and more specifically, hedonicity in relation to odors, such as the Affective Impact of Odor (AIO) scale (Wrzesniewski, 1999) or the Chemosensory Pleasure Scale (CPS) (Zhao et al., 2019). Nevertheless, these last two scales are not exactly focused on odor hedonicity. For example, the AIO measures the impact of odor on liking for places, people, foods, or cosmetics, in which, olfactory hedonicity is not explicitly assessed but through different sensory situations. Likewise, the CPS allows to assess the hedonic capacity for smell and taste pleasure, including both anticipatory and consummatory dimensions.

In light of the literature, it appears that the development of an odor hedonic scale is difficult. It is easy to rate a specific odor on a hedonic scale, but summing the ratings of several odors does not seem to be relevant because of the bipolar valence (pleasant/unpleasant) and the possible range of ratings (not at all/extremely). Moreover, hedonic responses to odors involve complex sensory, emotional, and cognitive processes (Wilson and Stevenson, 2006; Brand, 2020). Thus, it would be relevant to have a tool that breaks away from the sensory dimension since most of the olfactory hedonic variability comes from the stimulus and sniffing conditions or context. Finally, because the mental representation of odor is difficult and because of the specificity of odor memory related to the encoding context, the so-called Proust hypothesis is used (e.g., Chu and Downes, 2002; Herz and Schooler, 2002); this tool should offer a well-known activity or place associated with a significant odor (Chrea et al., 2005; Zarzo, 2008).

It is hypothesized that OHP can discriminate participants who overestimate or underestimate the hedonic rating of both pleasant and unpleasant odors. Thus, the OHP will allow to determine the extent to which it is possible to categorize subjects into different profiles with respect to their scores on all items.

Thus, this study aims to propose a tool to assess odor hedonicity, including three conditions, namely, without odorant stimulation, using a self-assessment, and quick to use (few minutes).

## 2. Material and methods

### 2.1. Pre-experiment

To select the relevant items to be included in the hedonic profile, a pre-experiment was conducted with a group of 30 participants (15 men and 15 women) based on 25 initial sentences/items. Consistent with previous studies on odor hedonic perception (e.g., Seubert et al., 2009 or Knaapila et al., 2017), each item was rated using a numerical scale, specifically ranging from −9 (“extremely unpleasant”) to +9 (“extremely pleasant”) through 0 (“neutral”) to account for hedonic tone variability in the hedonic ratings (see Table 1). Each dash of the line corresponds to a whole number (i.e., 1, 2, 3, …) in both positive and negative valences, and the space between two dashes corresponds to half a number (i.e., 0.5). In positive valence (between “neutral” and “extremely pleasant”), the first dash corresponds to +1, the second dash corresponds to +2, …, and the ninth dash corresponds to +9. The same notation is used in the negative valence (from neutral to extremely unpleasant). For example, if the participant places a cross in the negative valence on the third dash, the score of the item corresponds to −3. Similarly, if the participant places a cross in the negative valence between the third and fourth dash, the score of the item is equal to −3.5. When the cross is placed on the neutral dash, the score is 0.

**Table 1:** Odor hedonic profile model (OHP).

**1. When I take a walk in nature after a rainfall, I generally find the smell of rain:**	** 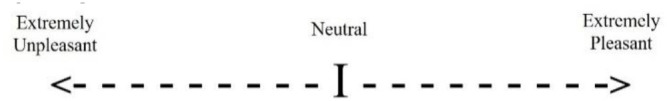 **
2. When I enter a hospital, I usually find the smell in the wards:	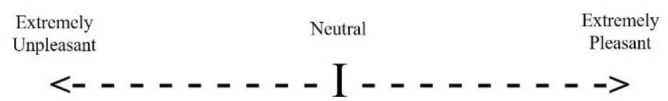
3. When I sweat after a physical effort, I generally find my odor:	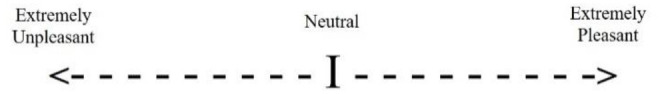
4. When I'm in a traffic jam, I usually find the smell of pollution:	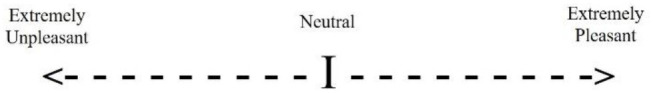
5. When I walk by the sea, I usually find the smell:	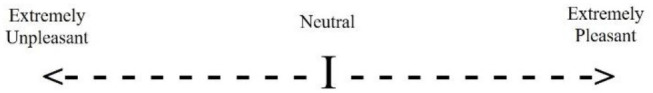
6. When I walk into a bakery, I usually find the smell of warm bread:	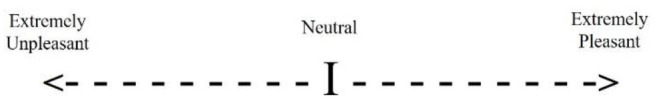
7. When I go to a gas station, I usually find the smell of fuel:	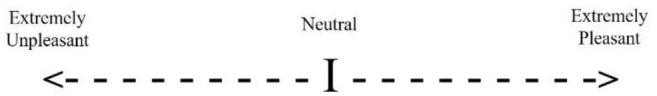
8. When I take public transportation (bus, tram, subway, etc.) during rush hour, I usually find the smell in the vehicle:	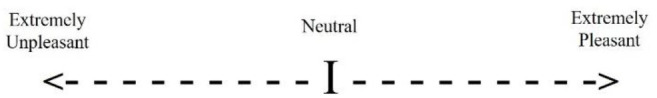
9. When I walk into a fish shop, I usually find the smell inside:	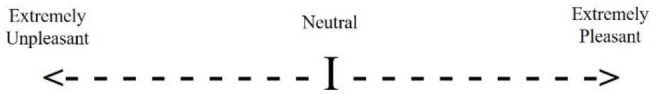
10. When I walk into a library/bookstore, I usually find the smell of books:	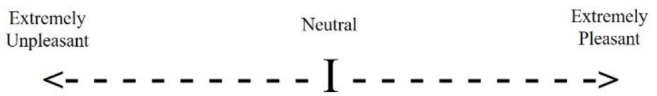
11. When I finish washing in the morning, I usually find my odor:	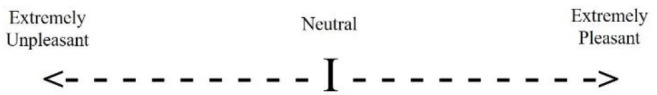
12. When I walk into a perfumery store, I usually find the smell inside:	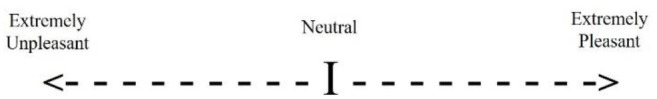
13. When I am grilling meat (or am close) to a barbecue, I usually find the smell:	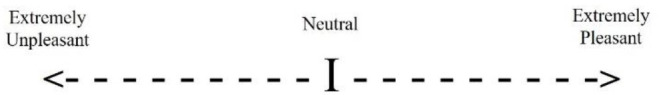
14. When I open the trash container to put my garbage, I usually find the smell:	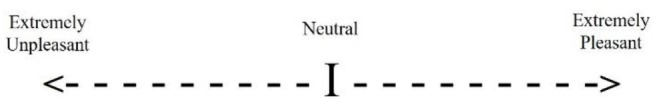

You will complete a 14-item questionnaire about the pleasant/unpleasant nature of odors in everyday situations. For each statement, please place a cross on the dotted line corresponding to the most accurate assessment of odor pleasantness between “neutral” and “extremely pleasant” or between “neutral” and “extremely unpleasant”.

Some of the items were excluded according to the following criteria. First, the distribution of scores for each item must follow a normal distribution. For example, the scores for the sentence “*When I talk with someone who smokes, I usually find the smell of cigarette*,” did not follow a normal distribution, so this item was excluded. Second, for each item, participants had to indicate whether they had ever experienced the situation. If more than two out of 30 participants (>5%) indicated that they had never been confronted with this context, the item was excluded as in the following case “*When I go to the swimming pool, I usually find the smell*.” Third, the place or the context must correspond to a *sui generis* smell (i.e., only one specific smell of the place or context). For example, the sentence “*When I go to a restaurant, I usually find the smell*,” was excluded because the odor depends strongly on the type of restaurant. Fourth, if two sentences presented a fairly close condition, the one with the most prominent odor was chosen. For example, the sentence “*When I walk into a fish shop, I usually find the smell inside*” was preferred to “*When I walk into a butcher's shop, I usually find the smell*.” Finally, a list of 14 items relating to food, leisure, health, washing, or transport was chosen to ask about the most common activities.

### 2.2. Main experiment

#### 2.2.1. Participants

To minimize sociocultural influences, the study was conducted in a homogeneous population of volunteer undergraduate students at the University of Franche-Comté (France).

*Inclusion criteria*: Participants must be between the ages of 19 and 26 years and enrolled in a first-degree psychology program. They must agree to participate in the study.

*Exclusion criteria*: Volunteers with diseases, such as nasal/sinus disorders, neurological and psychiatric disorders (e.g., schizophrenia and depressive or bipolar disorders), and eating disorders, were excluded. Volunteers who reported exposure to potentially toxic chemicals (including cigarette smoke) and those undergoing long-term medical treatment (excluding contraceptives) were also excluded from the study.

The study was conducted in accordance with the Declaration of Helsinki-Hong Kong as a result of which written informed consent was obtained from each participant prior to enrolment. The study design received no opposite statement from the Human Protection Committee East Area II (Besancon, France).

#### 2.2.2. Procedure

The experiment was carried out in a quiet room equipped with individual booths at the University of Franche-Comté. Upon arrival, participants gave written informed consent. They were then asked to fill out a personal information questionnaire: age, sex, smoking habits, and self-assessment of olfactory function on a scale from −9 (extremely bad smell perception) to +9 (excellent smell perception). They were then given instructions to complete the dashed line (Table 1) as follows: *You will complete a 14-item questionnaire about the pleasant/unpleasant nature of odors in everyday situations. For each statement, please place a cross on the dashed line corresponding to the most accurate rating of odor pleasantness between “neutral” and “extremely pleasant” or between “neutral” and “extremely unpleasant*.” As the present study was based on self-report, participants were unaware of the actual purpose of the study to avoid response bias, a concept well-known in psychological research (Orne, 1962; Orne and Whitehouse, 2000). The session lasted approximately 10/15 min. The score of each item was calculated in the same way as explained in the “Pre-experiment” section.

#### 2.2.3. Data analyses

The scores for each item were noted from −9 (extremely unpleasant) to +9 (extremely pleasant). For each item, the mean and the standard deviation were taken into account to determine the mean profile and the distribution centered around the mean. For each participant, the total score (TS) based on the 14 items was calculated in absolute value (i.e., from 0 to 126). The total of positive scores (T+) and the total of negative scores (T–) were also calculated, as well as the difference between (T+) and (T–) was calculated for each participant using total T+ and total T– scores.

Individual odor hedonic profile was determined using mean ± SD for TS, TS+, and TS– in order to have three ranges in each case, i.e., high, medium, and low. Liberal profile corresponded to high TS, T+, and T–; conservative profile corresponded to low TS, T+, and T–; neutral profile corresponded to medium TS, T+, and T–; positive olfactory alliesthesia profile corresponded to medium TS, high T+, and low T–; and negative olfactory alliesthesia profile corresponded to medium TS, low T+, and high T–.

Spearman's rank correlations were performed between scores for each item and (1) total scores (in absolute value), (2) total positive scores (T+), and (3) total negative scores (T–). In addition, Spearman's rank correlations were also performed between the self-reported smell score and (1) total scores (in absolute value), (2) total positive scores (T+), and (3) total negative scores (T–). Moreover, systematic comparisons in relation to sex were performed using independent samples Student's *t*-test. Bonferroni correction was applied to all data. The level of significance was *p* < 0.05.

## 3. Results

### 3.1. Participant's numbers and demographics

According to hedonic test recommendations, especially using a 9-point scale (Stone and Sidel, 2004), 100 participants took part in the main experiment (50 men and 50 women) (Mean age = 22.9 ± 1.4 years).

### 3.2. Hedonic profile

**Figure 1:**
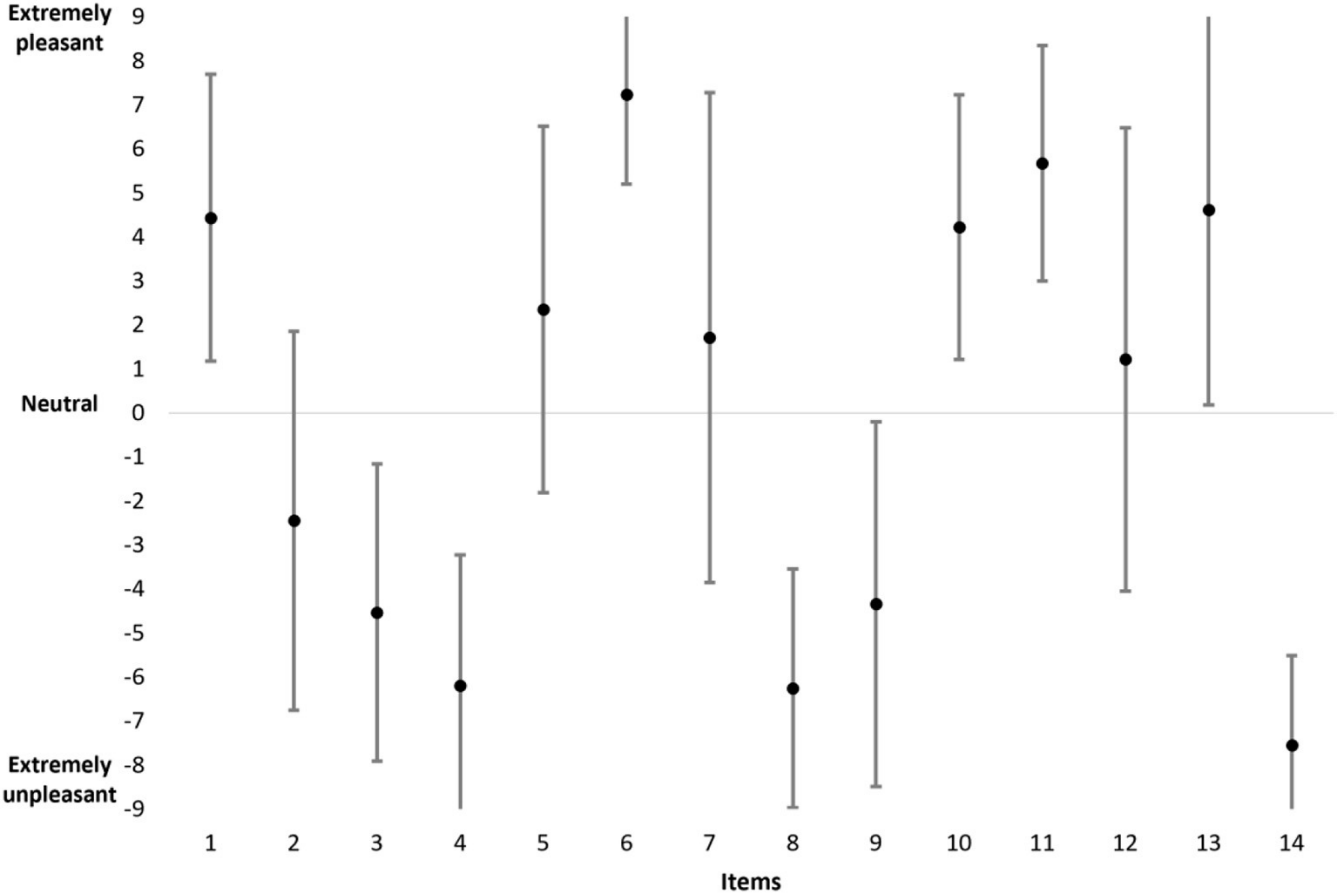
The mean and standard deviation of scores for all items in the entire population.

The results of the means and standard deviations are reported in Figure 1 for all items and for the whole population. The data reveal that the mean scores range from −7.54 (item 14) to +7.23 (item 6) and are evenly distributed along the dashed line. The data also show that the standard deviations vary between 2.03 (items 6 and 14) and 5.56 (item 7). The Spearman's rank correlation indicates a significant correlation between the mean (in absolute value) and the standard deviation (ρ = −0.853; *p* < 0.05). Thus, for an item, the higher the mean (T+ or T–) the lower the standard deviation (e.g., items 6 and 14) and vice versa (e.g., items 7 and 12).

**Figure 2:**
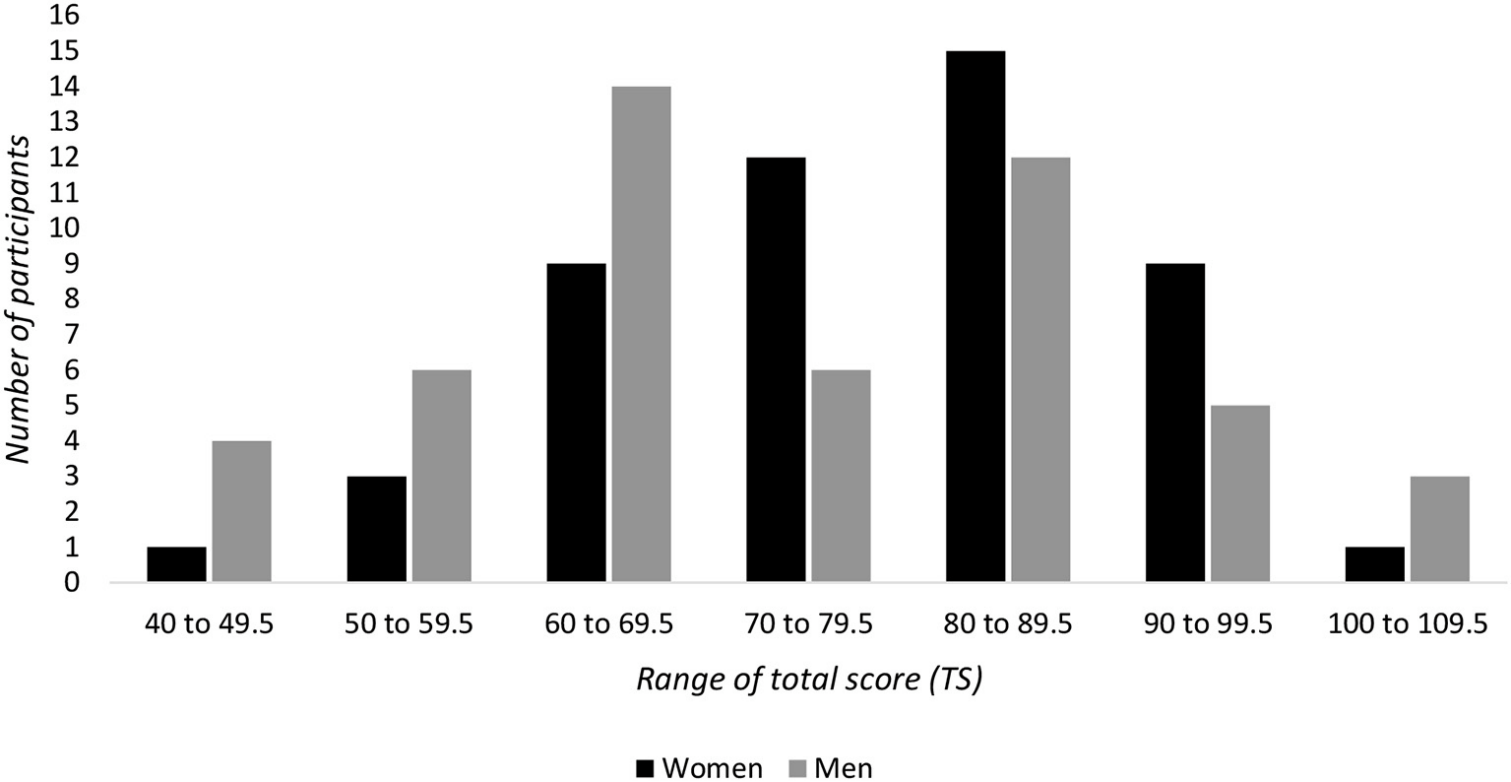
Total scores: number of participants for each total score range (e.g., 40 to 49.5 means that total score I comprised between 40 and 49.5 included). The total score (TS) is the absolute value of the sum of the hedonic scores of all items. Each item is rated on a dashed line ranging from −9 to 0 for unpleasant polarity and from 0 to +9 for pleasant polarity.

In absolute value, the total mean score based on the 14 items in the entire population is equal to 76.09 (sd = 14.59; minimum = 41; maximum = 105). Moreover, the results indicate the following values: the mean T+ = 38.02 (sd = 9.77; minimum = 16.5; maximum 61), the mean T– = −37.99 (sd = 11.94; minimum −64.5; maximum = −13), and the mean difference between T+ and T– = 0.045 (sd = 16.19; minimum = −43.5; maximum = 38). These data indicate a balanced distribution of the hedonic profile between positive and negative valences within the population. In addition, Figure 2 shows the number of participants in each total score range.

### 3.3. Correlations

The results of Spearman's rank correlations between scores for each item and total scores (in absolute value), between scores for each item and a total of positive scores (T+), and between each item scores and a total of negative scores (T–) are reported in Table 2. All item scores are at least correlated with one of the three scores (i.e., TS, T+, and T–). These data indicate that, in general, each participant rated most of the items in the same way, i.e., “strongly,” “moderately,” or “weakly.”

**Table 2:** Spearman's rank correlations between scores for each item and total scores (in absolute value) (TS), between scores for each item and a total of positive scores (T+), and between scores for each item and a total of negative scores (T–).

		**Scores item 1**	**Scores item 2**	**Scores item 3**	**Scores item 4**	**Scores item 5**	**Scores item 6**	**Scores item 7**	**Scores item 8**	**Scores item 9**	**Scores item 10**	**Scores item 11**	**Scores item 12**	**Scores item 13**	**Scores item 14**
TS	ρ	0.301	−0.285	−0.277	−0.403	0.026	0.474	0.135	−0.549	−0.501	0.344	0.440	−0.170	0.246	−0.415
	*p*	<0.01	<0.01	<0.01	<0.001	NS	<0.001	NS	<0.001	<0.001	<0.001	<0.001	NS	<0.05	<0.001
T+	ρ	0.261	0.06	0.174	−0.001	0.255	0.319	0.458	−0.141	0.022	0.327	0.445	0.245	0.465	−0.05
	*p*	<0.01	NS	NS	NS	<0.05	<0.001	<0.001	NS	NS	<0.001	<0.001	<0.05	<0.001	NS
T–	ρ	−0.165	0.383	0.487	0.478	0.171	−0.268	0.185	0.584	0.582	−0.162	−0.207	0.375	0.086	0.449
	*p*	NS	<0.001	<0.001	<0.001	NS	<0.01	NS	<0.001	<0.001	NS	<0.05	<0.001	NS	<0.001

Significant results were considered for *p* < 0.05. The term “NS” corresponds to a non-significant result.

The scores of 11 of the 14 items are correlated with TS, five positively and six negatively. Logically, the scores of 5 of the 14 items that are positively correlated with TS are positive (i.e., mean in the positive valence), and the scores of 6 of the 14 items that are negatively correlated with TS are negative (i.e., mean in the negative valence).

The scores of 8 of the 14 items are correlated with T+. These scores are positive (i.e., the mean has a positive valence), and the correlations are always positive.

The scores of 9 of the 14 items are correlated with T–, 7 positively and 2 negatively. The scores of 7 of the 14 items that are positively correlated are negative (i.e., mean in negative valence), except for the scores of items 6 and 12. On the other hand, the scores of two negatively correlated items are positive.

The results of the Spearman's rank correlations show no correlation between the estimate of smell score and any of the items' score, except for the score of item 11 (ρ = 0.296, *p* < 0.01), between the estimate of smell score and T+, between the estimate of smell score and T–, and between the estimate of smell score and T+/T– difference. In contrast, there is a significant correlation between TS and the estimate of smell score (ρ = 0.23, *p* < 0.05). This final result suggests that a participant with a low hedonic score reports a poor sense of smell and, conversely, a participant with a high hedonic score reports a good sense of smell.

### 3.4. Sex comparisons

**Figure 3:**
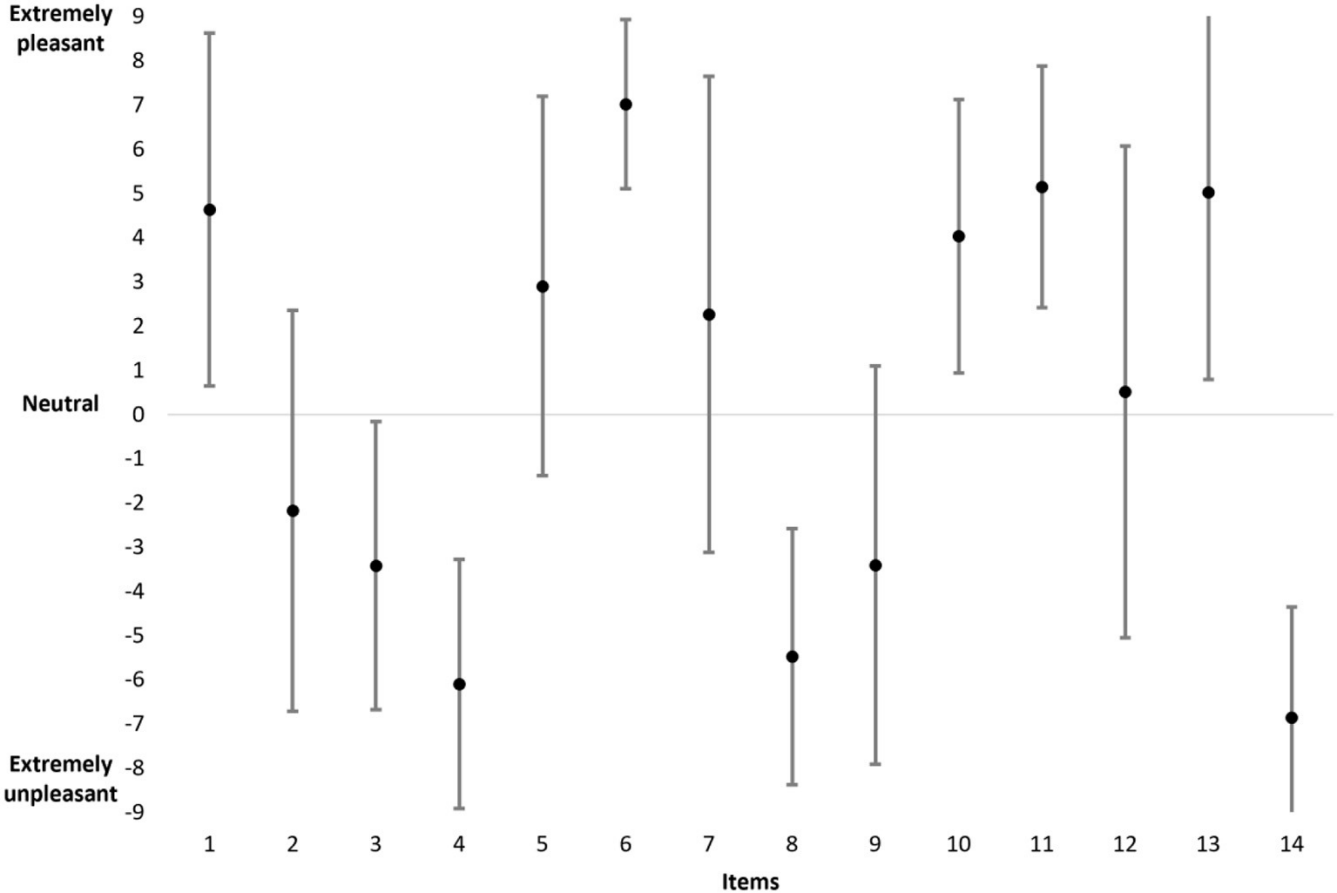
The mean and standard deviation of scores for all items in the men population.

For men (Figure 3), the data reveal that the mean scores range from −6.86 (item 14) to +7.02 (item 6) and are evenly distributed along the dashed line. The data also reveal that the standard deviations vary between 1.91 (item 6) and 5.56 (item 13). The Spearman's rank correlation indicates a significant correlation between the mean (in absolute value) and the standard deviation (ρ = −0.933, *p* < 0.001). Thus, for an item, the higher the mean (T+ or T–), the lower the standard deviation (e.g., items 6 and 14) and vice versa (e.g., items 7 and 12).

In absolute value, the total mean score based on the 14 items in the entire men population is equal to 73.53 (sd = 15.8; minimum = 41; maximum = 105). Moreover, the results indicate the following values: the mean T+ = 38.80 (sd = 9.93; minimum = 19; maximum 61), the mean T– = −34.73 (sd = 11.23; minimum −60; maximum = −18), and the mean difference between T+ and T– = 4.07 (sd = 14; minimum = −30; maximum = 38).

**Figure 4:**
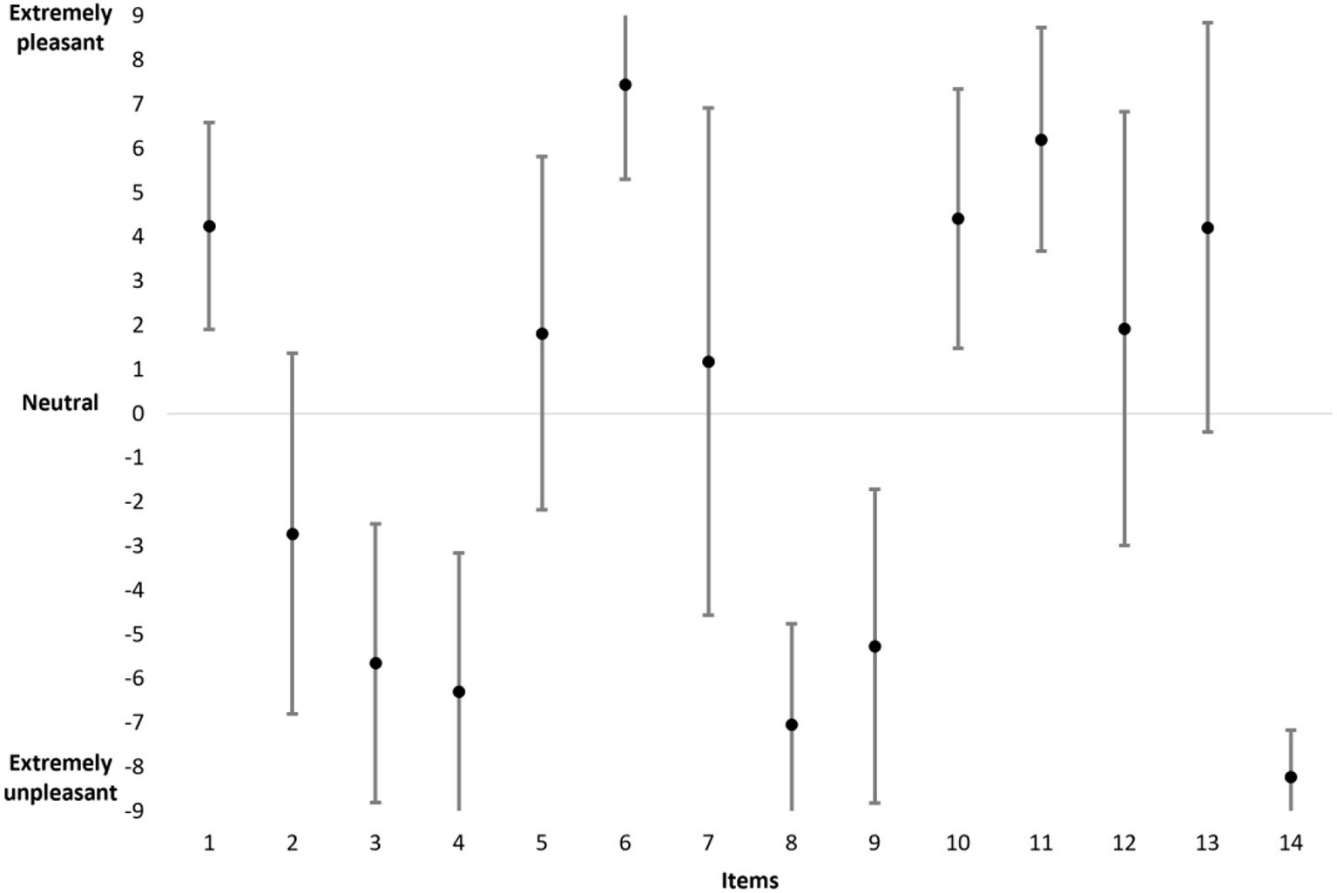
The mean and standard deviation of scores for all items in the women population.

For women (Figure 4), the data show that the mean scores range from −8.23 (item 14) to +7.44 (item 6) and are evenly distributed along the dashed line. The data also reveal that the standard deviations vary between 1.06 (item 14) and 5.74 (item 7). The Spearman's rank correlation indicates a significant correlation between the mean (in absolute value) and the standard deviation (ρ = −0.868, *p* < 0.001). Thus, for an item, the higher the mean (T+ or T–), the lower the standard deviation (e.g., items 6 and 14) and vice versa (e.g., items 7 and 12).

In absolute value, the total mean score based on the 14 items in the entire women population is equal to 78.65 (sd = 12.93; minimum = 49; maximum = 101.5). Moreover, the results indicate the following values: the mean T+ = 37.25 (sd = 9.65; minimum = 16.5; maximum 57), the mean T– = −41.25 (sd = 11.83; minimum −64.5; maximum = −13), and the mean difference between T+ and T– = −3.98 (sd = 14; minimum = −43.5; maximum = 29).

Systematic comparisons are conducted between men and women using independent *t*-tests and the results are reported in Table 3.

**Table 3:** Independent *t*-test comparison between men and women for each item score, for total score (TS), for a total of positive scores (T+), for a total of negative scores (T-), and for the difference between positive and negative scores (T+/T–).

	**Scores item 1**	**Scores item 2**	**Scores item 3**	**Scores item 4**	**Scores item 5**	**Scores item 6**	**Scores item 7**	**Scores item 8**	**Scores item 9**	**Scores item 10**	**Scores item 11**	**Scores item 12**	**Scores item 13**	**Scores item 14**	**TS**	**T+**	**T–**	**T+/T–**
Men (mean)	4.63	−2.18	−3.42	−6.10	2.90	7.02	2.26	−5.48	−3.41	4.03	5.15	0.51	5.02	−6.86	73.5	38.8	−34.7	4.1
Women (mean)	4.24	−2.72	−5.65	−6.3	1.81	7.44	1.17	−7.04	−5.27	4.41	6.20	1.92	4.21	−8.23	78.6	37.2	−41.2	−4.0
t	0.59	0.62	3.48	0.33	1.31	1.03	0.97	2.96	2.29	0.62	1.99	1.34	0.91	3.56	1.77	0.79	2.82	2.28
*p*	NS	NS	<0.001	NS	NS	NS	NS	<0.01	<0.05	NS	<0.05	NS	NS	<0.001	NS	NS	<0.01	<0.05

Significant results were considered for *p* < 0.05. The term “NS” corresponds to a non-significant result.

The total score comparison is neither significantly different between men and women nor is the T+ comparison. On the contrary, there is a significant difference for the T– comparison and for the T+/T– difference. These results indicate that men and women rate the items related to positive valence in the same way (only item 11 shows a significant difference), whereas women rate the items related to negative valence more negatively (significant difference for items 3, 8, 9, and 14).

### 3.5. Individual profiles

Different profiles emerge from individual data based on the mean ± sd criteria:

TS high > 90.68, TS medium between 90.68 and 61.50, and TS low <61.50.

T+ high > 47.79, TS medium between 47.79 and 28.25, and TS low <28.25.

T- high <−49.93, TS medium between −49.93 and −26.05, and TS low > −26.05.

**Figure 5:**
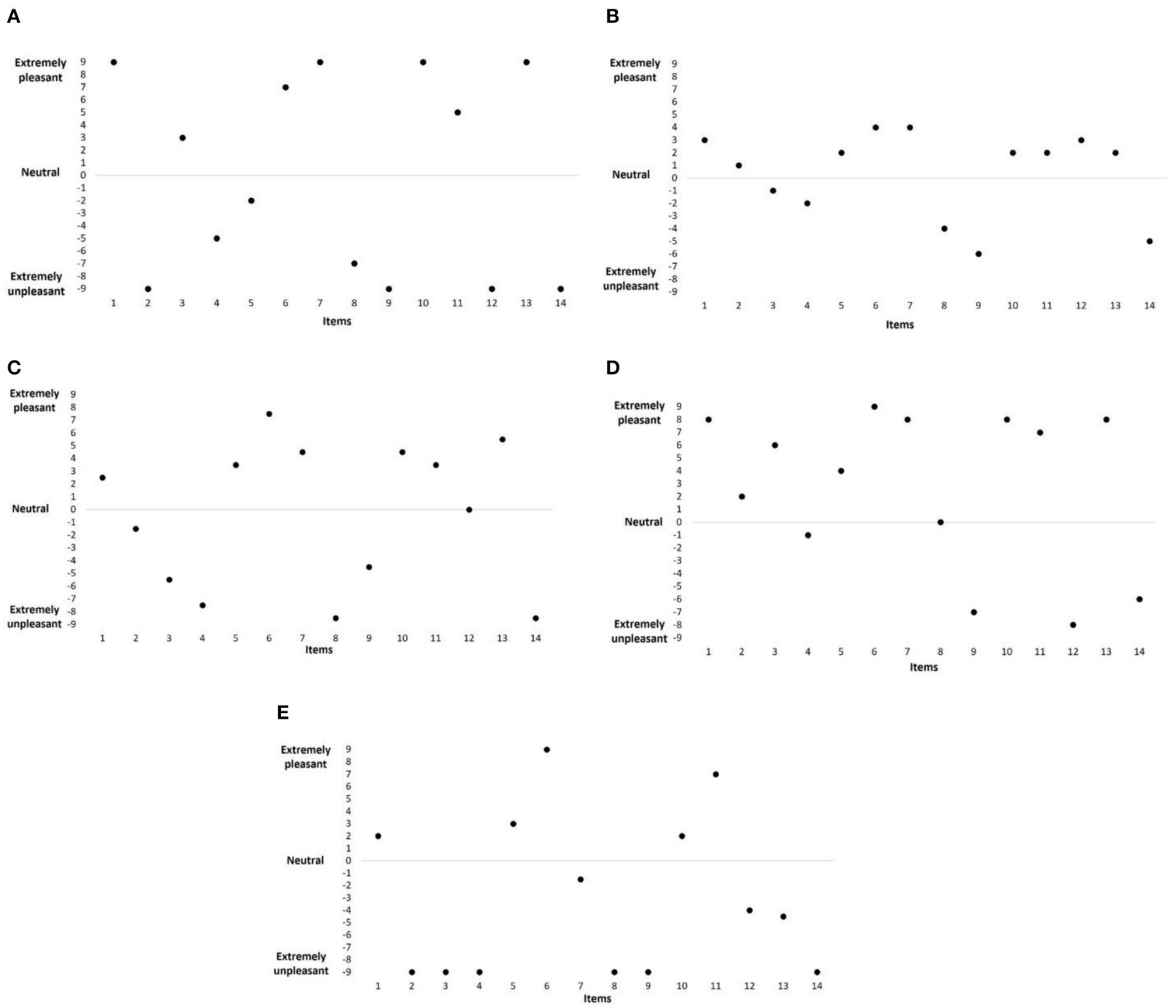
Example of individual profiles: scores for all items. **(A)** liberal profile (man); **(B)** conservative profile (man); **(C)** neutral profile (woman); **(D)** positive alliesthesia profile (man); and **(E)** negative alliesthesia profile (woman).

First, it can be distinguished as a *liberal profile* (Figure 5A) in which the participants give high scores in both cases of positive and negative valences and consequently get high TS, T+, and T– scores such as in the example (participant n°17, man) in Figure 5A: TS = 101; T+ = 51, and T– = −50. Second, it can be distinguished as a *conservative profile* (Figure 5B) in which the participants give low scores in both cases of positive and negative valences and consequently get low TS, T+, and T– scores such as in the example (participant n°11, man) in Figure 5B: TS = 41; T+ = 23, and T– = −18. Third, a *neutral profile* (Figure 5C) can be distinguished in which the participants give average scores in both cases of positive and negative valences and consequently get average TS, T+, and T– scores such as in the example (participant n°57, woman) in Figure 5C: TS = 67.5, T+ = 31.5, and T– = −36. Fourth, a *positive olfactory alliesthesia profile* (Figure 5D) can be distinguished in which the participants give high scores in positive valence and low scores in negative valence and consequently gets average TS, high T+, and low T– scores such as in the example (participant n°47, man) in Figure 5D: TS = 82, T+ = 60, and T– = −22. Fifth, a *negative olfactory alliesthesia profile* (Figure 5E) can be distinguished in which the participants give low scores in positive valence and high scores in negative valence resulting in average TS, low T+, and high T- scores such as (participant n°62, woman) in Figure 5E: TS = 87, T+ = 23, and T– = −64.

From the entire population (N = 100), the number of participants in each profile was as follows: *neutral profile* N = 37 (18 men and 19 women), *conservative profile* N = 13 (8 men and 5 women), *liberal profile* N = 10 (5 men and 5 women), *positive olfactory alliesthesia profile* N = 4 (2 men and 2 women), *negative olfactory alliesthesia profile* N = 5 (1 man and 4 women), and other profile N = 31 (15 men and 16 women).

## 4. Discussion

### 4.1. Key results

The results of this study showed a balanced distribution of the scores between positive and negative valences within the 14 items running along the axis from extremely unpleasant to extremely pleasant. Moreover, the scores of each item are normally distributed around the mean. The results also demonstrated that the OHP allows the determination of different specific profiles. Thus, with respect to the Detection Signal Theory (DST) first proposed by Tanner and Swets (1954) and later applied to psychophysics (Green and Swets, 1966), three profiles can be proposed, namely, “liberal,” “neutral,” and “conservative.” In the liberal profile, participants gave high ratings to both cases of positive and negative valences. Conversely, in the conservative profile, participants gave low ratings to both cases. These findings are also supported by a positive correlation between TS and self-rating of olfactory function. Many participants presented a “neutral profile” that seems consistent with the sample tested. In addition, the OHP was able to distinguish between positive and negative olfactory alliesthesia profiles. In olfaction, positive alliesthesia corresponds to rating pleasant odors as more pleasant and unpleasant odors as less unpleasant. Conversely, negative alliesthesia corresponds to rating pleasant odors as less pleasant and unpleasant odors as more unpleasant (Atanasova et al., 2010). Sex comparisons revealed specific differences for items related to negative valence. In fact, women rated the negative valence items significantly more negatively, whereas there was no sex difference for the positive valence items.

### 4.2. Limitations

From a limitation point of view, it is important to note that since the current data and classification are based on the mean and standard deviation of the group studied, this means that in another group of similar age and sex and without disease, the mean and standard deviation values may be slightly altered, resulting in a possible different distribution of values. However, the determination of the different profiles should be the same for two similar groups in terms of age, sex, and absence of disease. Similarly, hedonic estimate representation of odors could be subject to intra-individual variability and different factors, such as test time (e.g., morning vs. evening), hunger state (satiated or not), psychological state (e.g., stress level), or linked to the context (e.g., recent exposure to one or several items odors) may produce different results. As the present construct is not assumed to be extremely stable over time and aims to capture the actual state of participants, no test–retest was conducted.

Another limitation may be the lack of comparison between OHP scores and hedonic estimates using real odorants, which would allow determining the degree of agreement between the real olfactory perception and the representational perception. However, this comparison seems difficult given the number of variability parameters mentioned above. Likewise, the validation of the OHP should benefit from comparisons with different scales, i.e., odor scales, scales of psychological profiles, and questionnaires related to lifestyle, health, food consumption, etc.

### 4.3. Interpretation

From an interpretative point of view, the findings of this study showed two overlapping facts. Indeed, they confirmed the large interindividual variability in odor hedonic estimates and jointly revealed a general profile of the population with an equal ratio for the positive and negative valences. Moreover, this general profile presented a balanced structure with item scores evenly distributed along the dashed line from extremely unpleasant to extremely pleasant. This hierarchization in the classification according to hedonic estimation is consistent with the results of a previous study (Brand et al., 2012) and an adaptation of the Sniffin' Sticks Test, recently proposed to assess hedonic range (HR) and hedonic direction (HD) using 22 odorants (Liu et al., 2020). Finally, the results showed that the homogeneity of the scores depended on the item and clearly indicated that items with a high mean score (e.g., item 6 related to the bakery for positive valence and item 14 related to trash container for negative valence) had a small standard deviation and conversely for items with a low mean score and a large standard deviation covering both positive and negative valences. This suggests that the interindividual variability does not depend on the pleasant/unpleasant character of the odor but on the intensity of that character. Finally, although sex as an influencing factor of hedonic responses to odorants is poorly documented, these findings are consistent with some previous studies (Haidt et al., 1994; Broman and Nordin, 2000; Nordin et al., 2004; Olatunji et al., 2007; Luizza et al., 2017).

### 4.4. Generalizability

From a generalization point of view, this study is based on healthy undergraduate students, and few participants presented an olfactory alliesthesia profile, probably due to the homogeneous sample tested. The OHP may also be used to generally categorize individuals based on odor estimation in different populations such as in the elderly, in relation to personalities (e.g., introverted vs. extroverted), or in specific diseases. In the latter case, the OHP could be useful in individuals with eating disorders, as the specificity of alliesthesia to food cues has been previously demonstrated (Jiang et al., 2008). The OHP could also serve as a complementary diagnostic tool in conditions such as depressive states, insofar as olfaction is known to be strongly implicated in depression (Pause et al., 2001; Brand and Schaal, 2017). Using two odorants, one with pleasant (vanillin) and one with unpleasant (butyric acid) hedonic valence, a study (Atanasova et al., 2010) showed in depressed patients an olfactory negative alliesthesia (i.e., the unpleasant odorant was perceived as significantly more unpleasant than controls) and an olfactory anhedonia (i.e., impaired olfactory perception of the pleasant odorant). Another study (Clepce et al., 2010) showed that the relation between anhedonia and olfactory hedonics seems to be related to the severity of the disease. In contrast, patients with bipolar disorders rated odors to be more pleasant than healthy controls (Cumming et al., 2011), reflecting positive olfactory alliesthesia. In addition, hedonic ratings of pleasant odors may distinguish bipolar depression from unipolar depression (Kazour et al., 2020). This issue also arises in other pathologies, such as Parkinson's disease, for which specific hedonic tests using odorants have been proposed (Pospichalova et al., 2016) and anhedonia has been highlighted (Mrochen et al., 2016). Thus, the OHP could be usefully applied in clinical routines to avoid the use of odorants, thus helping to diagnose and monitor the evolution of the above-mentioned diseases. From a functional point of view, the OHP could be compared with specific anhedonia scales and further support the hypothesis that anhedonia (as in the case of the use of the International Affective Pictures Systems) refers to cognitive or emotional dysfunction rather than perceptual dysfunction, as in schizophrenia (Kamath et al., 2013; Atanasova et al., 2018). Finally, the OHP could be used in the context of odorant perception and odor-related studies (e.g., studies related to food odors). It may be of interest in future studies to compare profiles obtained from the OHP with hedonic ratings obtained after odor presentation.

## Data availability statement

The raw data supporting the conclusions of this article will be made available by the authors, without undue reservation.

## Ethics statement

Ethical approval was not provided for this study on human participants because CERTIFICATE Study name: Hedonic perception of odors in humans characterization of flexibility. BG and BC according to the French Regulatory Authority for clinical studies, prospective and retrospective studies with observation analysis only are not evaluated by Human Protection Committees. The study cited above had this study design and received no opposite statement from local Ethic Committee. Prof. Jean-Marc CHALOPIN, MD PhD President of the Human Protection Committee East Area II Besançon, France. The patients/participants provided their written informed consent to participate in this study.

## Author contributions

All authors listed have made a substantial, direct, and intellectual contribution to the work and approved it for publication.
